# Two-stage optimal designs with survival endpoint when the follow-up time is restricted

**DOI:** 10.1186/s12874-019-0696-x

**Published:** 2019-04-03

**Authors:** Guogen Shan, Hua Zhang

**Affiliations:** 1 Epidemiology and Biostatistics Program, Department of Environmental and Occupational Health, School of Community Health Sciences, University of Nevada Las Vegas, Las Vegas, 89154 NV USA; 2 School of Computer and Information Engineering, Zhejiang Gongshang University, Hangzhou, Zhejiang China

**Keywords:** Clinical trials, Exact variance, One-sample log-rank test, Restricted follow-up, Simon’s two-stage design

## Abstract

**Background:**

Survival endpoint is frequently used in early phase clinical trials as the primary endpoint to assess the activity of a new treatment. Existing two-stage optimal designs with survival endpoint either over estimate the sample size or compute power outside the alternative hypothesis space.

**Methods:**

We propose a new single-arm two-stage optimal design with survival endpoint by using the one-sample log rank test based on exact variance estimates. This proposed design with survival endpoint is analogous to Simon’s two-stage design with binary endpoint, having restricted follow-up.

**Results:**

We compare the proposed design with the existing two-stage designs, including the two-stage design with survival endpoint based on the nonparametric Nelson-Aalen estimate, and Simon’s two-stage designs with or without interim accrual. The new design always performs better than these competitors with regards to the expected total study length, and requires a smaller expected sample size than Simon’s design with interim accrual.

**Conclusions:**

The proposed two-stage minimax and optimal designs with survival endpoint are recommended for use in practice to shorten the study length of clinical trials.

## Background

A multiple-stage design is often preferable in early phase clinical trials to investigate the activity of a new treatment. Such design is able to protect patients better as compared to the traditional one-stage design by allowing a trial to be stopped earlier when the new treatment is indeed ineffective. For this reason, early stopping for futility is always allowed in these trials. Among multiple-stage designs, a two-stage design is widely used in phase II clinical trials whose sample size is relatively smaller than that in the following phase III trial to confirm the effectiveness of the new treatment(s).

When the outcome is binary (e.g., response VS non-response), Simon’s two-stage minimax and optimal designs are widely used in practice [1–8]. When the required number of patients in the first stage are enrolled, a trial generally has to be suspended temporally to allow these patients completing the treatment schedule. After that, data analysis is performed to make the decision whether a trial proceeds to the second stage or not, based on the result from the first stage. This suspension during the clinical trial could lead to a longer study time as compared to the modified Simon’s two-stage design with interim accrual [9]. Recently, adaptive version of Simon’s two-stage design has been proposed to improve the flexibility of trials [3, 4, 10–12]. In such trials, the second stage sample size depends on the outcome from the first stage.

In some other trials (e.g., cytostatic therapies), a survival endpoint is served as the primary outcome to measure the activity of a new treatment. Feldman et al. [13] reviewed seven single-arm phase II trials for patients with refractory germ cell tumors, and recommended a 12-week progression-free survival as compared to the commonly used response rate, to test the activity of novel agents. For such trials, a multiple-stage design with survival endpoint would be appropriate for use in practice. Lin et al. [14] proposed group sequential designs for a trial with survival endpoint by deriving the asymptotic joint distribution of the Nelson-Aalen estimates at different time points. Base on Lin et al.’s work, Case and Morgan [9] developed a two-stage optimal design evaluating survival probabilities with restricted follow-up. They proposed two-stage optimal designs with the smallest expected duration of accrual or the smallest expected total study length. Later, Kwak and Jung [15] proposed a new two-stage optimal design based on the one-sample log-rank test without follow-up restriction. Power of their proposed design was computed under the average of the cumulative hazard function under the null hypothesis and that under the alternative hypothesis. In addition, the asymptotic variance estimate of the one-sample log-rank test was used in type I error rate and power calculation. Recently, Belin et al. [16] proposed a two-stage design based on the design setting as in Kwak and Jung [15], but having restricted follow-up as in Case and Morgan [9].

For a trial with a survival endpoint as the primary outcome, the survival probability at the clinically meaningful follow-up time is often the parameter of interest, (e.g., the survival probability at 1 year). We develop a new single-arm two-stage optimal design by using the one-sample log-rank test with exact mean and variance estimates [17, 18]. A trial is allowed to be stopped in the first stage due to futility to protect patients when the treatment under investigation is indeed ineffective. Although exact mean and variance estimates of the one-sample log-rank test are used for sample size calculation, the joint distribution of the test statistic for the first stage and that for the two stages combined is assumed to asymptotically follow a bivariate normal distribution. For this reason, the actual power of the identified study design may not be guaranteed [19]. We propose adjusting the nominal power level in design search to guarantee that the new designs meet the power requirement. The proposed two-stage minimax and optimal designs with survival endpoint are compared with the design by Belin et al. [16] and Simon’s two-stage designs with or without interim accrual.

The rest of this article is organized as follows. In Section Methods, we present the type I error rate and power calculation for a two-stage design with survival endpoint by using the one-sample log-rank test, and provide a detailed search method for two-stage minimax and optimal designs. In Section Results, we compare the performance of the new proposed two-stage designs with the existing Belin’s design with survival endpoint and Simon’s two-stage design with binary endpoint. At the end of that section, we revisit two trials to illustrate the application of the proposed two-stage designs with survival endpoint. Lastly, we provide some comments in Section Discussion.

## Methods

Suppose *S*(*t*) is the survival function of the survival time *T*. In a single-arm study, the survival probability of a new treatment at the clinically meaningful follow-up time *t*_*c*_, *S*(*t*_*c*_), is compared to the estimated historical survival probability, *S*_0_(*t*_*c*_). Then the hypotheses are presented as

1 $$ H_{0}: S(t_{c})\leq S_{0}(t_{c}) \ \ \text{against} \ \ H_{1}: S(t_{c})> S_{0}(t_{c}).   $$

In this article, the survival function *S*(*t*) is assumed to follow the Weibull distribution with the shape parameter *k* and the scale parameter *λ*, specifically,

$$S(t)=\exp^{-(t/\lambda)^{k}},$$

where *k*>0 and *λ*>0. The widely used exponential distribution is a special case of the Weibull distribution when *k*=1.

Under the Weibull distribution for survival outcome, suppose the failure rate under the null hypothesis is the same as that under the alternative hypothesis (the same shape parameter *k*), but scale parameters are different with *λ*_0_ and *λ*_1_ under the null hypothesis and the alternative hypothesis, respectively. Then, *Δ*=(*λ*_0_/*λ*_1_)^*k*^ is the hazard ratio (HR), which is always less than 1 under the alternative. The hypotheses in Eq. (1) can be specifically rewritten as

2 $$ H_{0}: \Delta\geq 1 \ \ \text{against} \ \ H_{1}: \Delta<1.   $$

When a new study is assumed to have a different failure rate as historical data, the HR is then calculated as $\Delta =\frac {\lambda _{0}^{k_{0}}}{\lambda _{1}^{k_{1}}} \times \frac {k_{1} t^{k_{1}-1}}{k_{0} t^{k_{0}-1}}$, where *k*_0_ and *k*_1_ are the shape parameter under the null hypothesis and that under the alternative hypothesis, respectively.

### Simon’s two-stage designs with binary endpoint

In Simon’s two-stage optimal designs, a trial is allowed to be stopped in the first stage when the number of responses is insufficient. Suppose *X*_1_ and *X* are the number of responses out of *n*_1_ and *n* participants from the first stage and the two stages combined, respectively. The sample size in the second stage is *n*_2_=*n*−*n*_1_. The null hypothesis is rejected when *X*_1_>*r*_1_ and *X*>*r*, where *r*_1_ and *r* are the critical values for the number of responses from the first stage and both stages, respectively.

In a pancreatic cancer trial with a combination of Gemcitabine and external beam radiation as the new treatment [9], the clinically meaningful follow-time is 1 year, *t*_*c*_=1. The unacceptable one-year survival rate is *S*_0_(1)=35*%*, and the new treatment is considered as promising for further investigation when *S*_1_(1)=50*%* or more. To attain 90% power of the study at the significance level of 10%, Simon’s two-stage minimax design [1] is calculated as:

$$(n_{1},r_{1},n,r)=(43,14,72,30),$$

with the expected sample size under the null hypothesis *ESS*_0_=*n*_1_+(1−*PET*)*n*_2_=59.3, where *PET* is the probability of early termination under the null hypothesis which is defined as *PET*=*p*(*X*_1_≤*r*_1_|*S*_0_(1)=35*%*)=43.65*%*. Suppose this is a 3 year study with the patient accrual rate of *θ*=24 patients per year. Then the enrollment time for the first stage and the second stage is calculated *t*_1_=*n*_1_/*θ* and *t*_2_=*n*_2_/*θ*, respectively. The expected total study length (ETSL) under the null hypothesis is calculated as

$${ETSL}_{0}=(t_{1}+t_{c})+(1-PET)(t_{2}+t_{c})=4.0 \ \text{years}$$

The two-stage optimal design needs *ESS*_0_=53.2 and *ETSL*_0_=3.6 years (see Table 1). The maximum possible sample size for Simon’s optimal design *n*=81 is much larger than *n*=72 for Simon’s minimax design.

**Table 1:** The resectable pancreatic cancer clinical trial with *S*_0_(*t*_*c*_=1)=35*%*, and *S*_1_(*t*_*c*_=1)=50*%* to attain 90% power at the significance level of 10%

	Survival endpoint								Simon’s design, interim accrual				
	The proposed method						Belin		No			Yes	
	*n*_1_	*n*	*c*_1_	*c*	*E* *S* *S*_0_	*E* *T* *S* *L*_0_	*E* *S* *S*_0_	*n*	*E* *S* *S*_0_	*E* *T* *S* *L*_0_	*n*	*E* *S* *S*_0_	*E* *T* *S* *L*_0_
Minimax	44	73	0.240	-1.281	61.3	3.1			59.3	4.0	72	69.8	3.5
Optimal	41	79	-0.085	-1.279	58.7	2.9	59.1	69	53.2	3.6	81	67.4	3.2

The survival function follows an exponential distribution

When Simon’s two-stage design allows interim accrual at the end of the first stage, the expected sample size under the null hypothesis is calculated as

$${ESS}_{0}=n_{1}+\theta t_{c} +(1-PET) (n_{2}-\theta t_{c}),$$

and the expected total study length under the null hypothesis is

$${\begin{aligned} {ETSL}_{0}&=(t_{1}+t_{c})+(1-PET) \left[(t_{2}-t_{c})+t_{c}\right]\\&\quad=(t_{1}+t_{c})+(1-PET) t_{2} \end{aligned}} $$

The results of Simon’s two-stage designs with interim accrual are presented in Table 1. As compared to the traditional Simon’s two-stage design without interim accrual, the modified design with interim accrual requires a shorter *ETSL*_0_ but a larger *ESS*_0_.

### Two-stage optimal designs with survival endpoint when the follow-up time is limited

In a two-stage design with sample sizes of *n*_1_ in the first stage and *n*_2_ in the second stage, the maximum possible sample size in the study is *n*=*n*_1_+*n*_2_. Given the patient accrual rate of *θ*, the accrual time for the first stage is *t*_1_=*n*_1_/*θ*. When the trial goes to the second stage, the total accrual time of the study is *t*_*a*_=*n*/*θ*, and the total study time for all patients to complete the study is *t*=*t*_*a*_+*t*_*c*_.

We assume that patients are uniformly enrolled in the study, with the entering times of *τ*_1_,*τ*_2_,⋯,*τ*_*n*_. They have the survival times of *T*_1_,*T*_2_,⋯,*T*_*n*_ and the censoring times of *C*_1_,*C*_2_,⋯,*C*_*n*_. At the end of the first stage *t*_1_, the observed time for the *i*-th patient is the smallest of the following three measurements: (1) event time; (2) censoring time; and (3) time that this patient is followed so far in the study, specifically,

$$O_{i}=\min(T_{i}, C_{i}, \max(0,t_{1}-\tau_{i})).$$

By using the observed time and the censoring information of the first *n*_1_ patients, the one-sample log-rank test can be calculated as

$$Z_{1}=\frac{W_{1}}{\hat\sigma_{1}},$$

where *W*_1_ is a function of the difference between observed number of events and the expected number of events, and $\hat \sigma _{1}$ is its standard deviation estimate. Please find the detailed formula of *Z*_1_ under the null hypothesis and the alternative hypothesis in Appendix.

The null hypothesis is rejected when a small test statistic is observed. Suppose the critical value for *Z*_1_ is *c*_1_. When the calculated *Z*_1_ is larger than or equal to *c*_1_, the trial is stopped for futility and no further investigation is warranted. Otherwise, the trial goes to the second stage with additional *n*_2_=*n*−*n*_1_ patients treated by the new treatment. At the end of study when all *n* patients complete the study, the one-sample log-rank test is calculated as

$$Z=\frac{W}{\hat\sigma}.$$

It can be seen that *Z*_1_ and *Z* are not independent from each other since the data of the first *n*_1_ patients is used in both *Z*_1_ and *Z*. The type I error (TIE) rate of the study is calculated as

$$TIE=P(Z_{1}\leq c_{1}, Z\leq c | H_{0}),$$

where *c* is the critical value for *Z*.

Following Kwak and Jung [15], the joint distribution of (*Z*_1_,*Z*) is a bivariate normal distribution asymptotically. Then, the TIE can be specifically written as

3 $$ TIE=\int_{-\infty}^{c} \phi(t) \Phi\left(\frac{c_{1}-\rho_{0} t}{\sqrt{1-\rho_{0}^{2}}}\right) d t,   $$

where *ϕ* and *Φ* are the probability density function and the cumulative distribution function of the standard normal distribution, and *ρ*_0_ is the correlation coefficient estimate between *Z*_1_ and *Z* under the null hypothesis, see Appendix for the detailed formula for *ρ*_0_. The actual power of the study can be computed similarly with *ρ*_0_ being replaced by the *ρ* estimate under the alternative hypothesis.

#### Optimal design search

Similar to the search for Simon’s two-stage design, the two-stage optimal design with survival endpoint has to be searched over all the possible sample sizes (*n*_1_ and *n*) and critical values (*c*_1_ and *c*), given the design parameters (*α*,*β*,*t*_*c*_,*S*_0_(*t*_*c*_),*S*_1_(*t*_*c*_),*θ*).

Although the exact variances of *Z*_1_ and *Z* are available for use in sample size determination, the exact joint distribution of *Z*_1_ and *Z* is not that straightforward. For this reason, we utilize the limiting distribution of (*Z*_1_,*Z*) in searching for the two-stage optimal design for a study with the design parameters (*α*,*β*,*t*_*c*_,*S*_0_(*t*_*c*_),*S*_1_(*t*_*c*_),*θ*), then use a simulation study to calculate the actual TIE and power of the optimal design. The following three steps are used to search for the two-stage minimax and optimal designs.

**Step 1**: Given the total sample size *n*, the range of the first stage sample size *n*_1_ is from 1 to *n*−1. The critical value *c*_1_ from -0.3 to 1.6 with an increment of 0.005 is used in the design search. Similar to Kwak and Jung [15], the range of *c*_1_ is chosen based on the simulation studies for all the configurations studied in this article. The range of *c*_1_ is modifiable in the software program for design search.

For each combination of *n*_1_ and *c*_1_, the critical value *c* can be determined as the largest *c* value such that *TIE*(*c*)≤*α* from Eq. (3). Power of the study is then computed by using Eq. (4) in Appendix. If power is above the nominal level, this set of sample sizes and critical values, (*n*_1_,*c*_1_,*n*,*c*), is saved as a candidate for the optimal two-stage design. Among all the sets satisfying the power requirement, the one with the smallest *ESS*_0_ is the optimal two-stage design when the total sample size is *n*, and it is denoted as *B*(*n*)=(*n*_1_,*c*_1_,*n*,*c*) whose expected sample size is *ESS*_0_(*n*).

**Step 2**: The design search starts with a relatively small *n* (e.g., 5) with an increment of 1, and *B*(*n*) could be a empty set when *n* is small. The two-stage minimax design is the one with the smallest *n*, *n*_*minimax*_ such that *B*(*n*) is not empty. The optimal two-stage design is the one with the smallest *ESS*_0_. The search may be stopped at *n*_*u*_ when its *ESS*_0_(*n*_*u*_) is 10% more than the smallest *ESS*_0_ from the identified optimal designs with *n* from *n*_*minimax*_ to *n*_*u*_: *ESS*_0_(*n*_*u*_)≥110*%*× min{*ESS*_0_(*n*):*n*_*minimax*_≤*n*≤*n*_*u*_}.

**Step 3**: Once the minimax and optimal two-stage designs are identified from Step 1 and Step 2, we use a simulation study to calculate the actual TIE and power based on 100,000 simulations. We find that the actual TIE of the optimal design *B*(*n*)=(*n*_1_,*c*_1_,*n*,*c*) is always guaranteed, while power may not be preserved in some cases. If the simulated power of the two designs meet the nominal levels, they are the final two-stage minimax and optimal designs. Otherwise, we search for the designs again with the power nominal level being increased by 1%, (*α*,*β*−1*%*) in Step 1 and Step 2 again. This process is stopped when both minimax and optimal two-stage designs meet the power requirement.

## Results

We first compare the proposed two-stage minimax and optimal designs with survival endpoint when the follow-up time is restricted, with the designs developed by Belin et al. [16] (referred to as Belin’s design). They developed a two-stage optimal design as a modification of the design by Kwak and Jung [15] by adding restricted follow-up in the study design [9]. In Belin’s design, power of the study is computed at the average of the cumulative hazard functions under the null and the alternative, that is less than the cumulative hazard functions under the alternative at which value the actual power should be computed. This leads to an decreased effect size in sample size calculation; thus, the computed sample size may be over-estimated. As a result of the over-estimated sample size, the actual power is often above the nominal level.

Table 2 shows the comparison between the proposed designs with Belin’s design, when the survival distribution follows an exponential distribution. Belin et al. [16] investigated the performance of two-stage optimal designs with restricted follow-up under exponential distributions only (the shape parameter *k*=1 in the Weibull distribution). The clinically meaningful follow-up time *t*_*c*_ is assumed to be 1 year. Under the null hypothesis, the survival rate at *t*_*c*_=1 is *S*_0_(*t*_*c*_)=50*%* (*λ*_0_=1.44) as studied in Table 2. The hazard ratio is assumed to be 0.5, which is *Δ*=*λ*_0_/*λ*_1_=0.5. Then the scale parameter under the alternative is *λ*_1_=2.88. The nominal power level is set as either 90% or 95%. The accrual rate *θ* is 15, 30, or 50. The *ESS*_0_ of the proposed minimax or optimal designs is often less than that of the Belin’s design, that may be due to the fact that power of Belin’s design is computed outside the alternative hypothesis space. The simulated TIE and power of the developed two-stage minimax and optimal designs are shown in Table 3. In Table 3, we also report the 95% confidence interval for the TIE and power based on 1,000 simulated TIE and power values, where each simulated TIE and power are computed using 10,000 simulations. It can be seen that the proposed designs control for TIE and power.

**Table 2:** Comparison between the proposed two-stage minimax and optimal designs with survival endpoint and Belin’s two-stage optimal design with survival endpoint, when the follow-up time is restricted to the clinically meaningful follow-up time *t*_*c*_=1 year

		Minimax design					Optimal design					Belin	
Power	*θ*	*n*_1_	*n*	*c*_1_	*c*	*E* *S* *S*_0_	*n*_1_	*n*	*c*_1_	*c*	*E* *S* *S*_0_	*n*	*E* *S* *S*_0_
90%	15	28	52	-0.10	-1.64	39.1	26	56	-0.30	-1.64	37.5	53	42.3
95%	15	36	65	-0.09	-1.64	49.5	33	70	-0.29	-1.64	47.3	65	52.6
90%	30	30	52	0.30	-1.64	43.6	30	55	-0.04	-1.64	42.2	53	44.6
95%	30	40	65	0.19	-1.64	54.3	40	69	-0.20	-1.64	52.2	65	54.8
90%	50	34	52	0.51	-1.64	46.5	32	54	0.32	-1.63	45.7	52	47.0
95%	50	44	65	0.46	-1.64	58.2	42	68	0.17	-1.64	56.7	64	57.5

The null survival probability at 1 year is *S*_0_(*t*_*c*_)=50*%*, and the hazard ratio is 2. Patient accrual rate *θ* is set as 15, 30, or 50 per year

**Table 3:** Simulated TIE and power of the proposed two-stage minimax and optimal designs in Table 2

		Minimax design		Optimal design	
Power	*θ*	TIE	Power	TIE	Power
90%	15	0.040 (0.036,0.044)	0.907 (0.901,0.913)	0.037 (0.033,0.041)	0.903 (0.898,0.909)
95%	15	0.041 (0.037,0.045)	0.957 (0.953,0.961)	0.038 (0.035,0.042)	0.955 (0.951,0.959)
90%	30	0.040 (0.037,0.044)	0.911 (0.905,0.916)	0.039 (0.035,0.043)	0.910 (0.904,0.916)
95%	30	0.042 (0.038,0.046)	0.959 (0.955,0.963)	0.040 (0.036,0.044)	0.958 (0.954,0.962)
90%	50	0.041 (0.037,0.045)	0.911 (0.905,0.916)	0.040 (0.037,0.044)	0.909 (0.903,0.914)
95%	50	0.042 (0.038,0.046)	0.960 (0.956,0.963)	0.041 (0.037,0.045)	0.959 (0.955,0.963)

The 95% confidence intervals for the parameters of interest are computed using 1000 simulations where 10,000 designs are simulated in each simulation

We further compare the proposed two-stage minimax and optimal designs with survival endpoint, with Simon’s two-stage designs with or without interim accrual for a trial with binary endpoint, see Table 4 when the survival distribution follows the Weibull distribution with a common shape parameter of *k*=0.5. The significance level is set as 5%, and the nominal power level is 80%. The null survival probability at the clinically meaningful follow-up time *t*_*c*_=1, *S*_0_(*t*_*c*_)=10*%* and 60% are studied in Table 4. We consider a medium to large effect size as *S*_1_(*t*_*c*_)−*S*_0_(*t*_*c*_)= 10%, 15%, and 20%. For each configuration of *S*_0_(*t*_*c*_) and *S*_1_(*t*_*c*_), the scale parameters *λ*_0_ and *λ*_1_ in the Weibull distribution can be calculated, the *ESS*_0_ and *ETSL*_0_ of the proposed minimax design and Simon’s minimax design are computed. Patient accrual rate *θ* is calculated by assuming it is a 3 year study when Simon’s two-stage minimax design is used. In the table, percentage (%) is for the *ESS*_0_ or the *ETSL*_0_ percentage saving of the proposed two-stage design with survival endpoint as compared to Simon’s two-stage design, which is computed as (Simon-New)/Simon. When the percentage saving is positive, the new design requires a smaller *ESS*_0_ or a shorter *ETSL*_0_ as compared to the existing Simon’s design. When the null survival probability *S*_0_(*t*_*c*_) is low, say 10%, the proposed two-stage design with survival endpoint saves sample size as compared to Simon’s two-stage minimax design. This trend is reversed when *S*_0_(*t*_*c*_)=60*%*. In Table 4, we also present the results of Simon’s two-stage minimax design with interim accrual. It can be seen that the new design always requires a smaller *ESS*_0_ than Simon’s design with interim accrual. The new design always saves the *ETSL*_0_ as compared to Simon’s design with or without interim accrual. The saving becomes smaller as the null survival probability goes up from 10% to 60%. Similar results are observed in Table 5 for the two-stage optimal designs.

**Table 4:** Comparison between the proposed two-stage minimax design with survival endpoint and Simon’s two-stage minimax design with binary endpoint with or without interim accrual, when *α*=5*%*, *β*=20*%*, and the shape parameter *k*=0.5 in the Weibull distribution

						Simon’s two-stage minimax designs					
		Survival endpoint				No interim accrual				Interim accrual	
*S*_0_(*t*_*c*_)	*S*_1_(*t*_*c*_)	*n*_1_	*n*	*E* *S* *S*_0_	*E* *T* *S* *L*_0_	*n*_1_	*n*	*ESS*_0_(*%*)	*ETSL*_0_(*%*)	*ESS*_0_(*%*)	*ETSL*_0_(*%*)
0.1	0.2	37	63	50.5	2.5	45	78	60.6 (17%)	3.8 (35%)	74.3 (32%)	3.3 (26%)
0.1	0.25	19	33	26.2	2.5	22	40	28.8 (9%)	3.5 (30%)	37.5 (30%)	3.1 (21%)
0.1	0.3	11	21	15.6	2.3	15	25	19.5 (20%)	3.8 (39%)	24.5 (36%)	3.3 (30%)
0.6	0.7	87	162	126.6	3.2	139	142	139.2 (9%)	4.0 (20%)	184.5 (31%)	3.9 (19%)
0.6	0.75	33	70	49.4	2.8	30	62	43.8 (-13%)	3.6 (20%)	55.7 (11%)	3.1 (9%)
0.6	0.8	17	39	26.0	2.6	13	35	20.8 (-25%)	3.1 (16%)	28.5 (9%)	2.8 (5%)

% is for the *ESS*_0_ or the *ETSL*_0_ percentage saving of the new proposed two-stage design as compared to Simon’s two-stage design, which is computed as (Simon-New)/Simon. When the percentage saving is positive, the new design requires a smaller *ESS*_0_ or a shorter *ETSL*_0_ as compared to the existing Simon’s design

The patient accrual rate *θ* is determined by the sample size from Simon’s minimax design with no interim accrual as *θ*=*n*_*minimax*_/3

**Table 5:** Comparison between the proposed two-stage optimal design with survival endpoint and Simon’s two-stage optimal design with binary endpoint with or without interim accrual, when *α*=5*%*, *β*=20*%*, and the shape parameter *k*=0.5 in the Weibull distribution

						Simon’s two-stage optimal designs					
		Survival endpoint				No interim accrual				Interim accrual	
*S*_0_(*t*_*c*_)	*S*_1_(*t*_*c*_)	*n*_1_	*n*	*E* *S* *S*_0_	*E* *T* *S* *L*_0_	*n*_1_	*n*	*ESS*_0_(*%*)	*ETSL*_0_(*%*)	*ESS*_0_(*%*)	*ETSL*_0_(*%*)
0.1	0.2	26	72	45.1	2.2	30	89	50.8 (11%)	3.3 (35%)	67.6 (33%)	3.0 (27%)
0.1	0.25	15	37	24.0	2.2	18	43	24.7 (3%)	3.1 (29%)	34.9 (31%)	2.8 (22%)
0.1	0.3	10	23	15.0	2.2	10	29	15.0 (0%)	3.1 (29%)	21.6 (30%)	2.8 (21%)
0.6	0.7	66	179	109.2	2.7	53	173	91.4 (-20%)	3.3 (18%)	124.0 (12%)	2.9 (9%)
0.6	0.75	27	76	46.1	2.6	27	67	39.4 (-17%)	3.2 (18%)	53.9 (14%)	2.9 (10%)
0.6	0.8	15	41	25.1	2.5	11	43	20.5 (-23%)	3.1 (17%)	28.9 (13%)	2.8 (7%)

% is for the *ESS*_0_ or the *ETSL*_0_ percentage saving of the new proposed two-stage design as compared to Simon’s two-stage design, which is computed as (Simon-New)/Simon. When the percentage saving is positive, the new design requires a smaller *ESS*_0_ or a shorter *ETSL*_0_ as compared to the existing Simon’s design

The patient accrual rate *θ* is determined by the sample size from Simon’s minimax design with no interim accrual as *θ*=*n*_*minimax*_/3

**Fig. 1:**
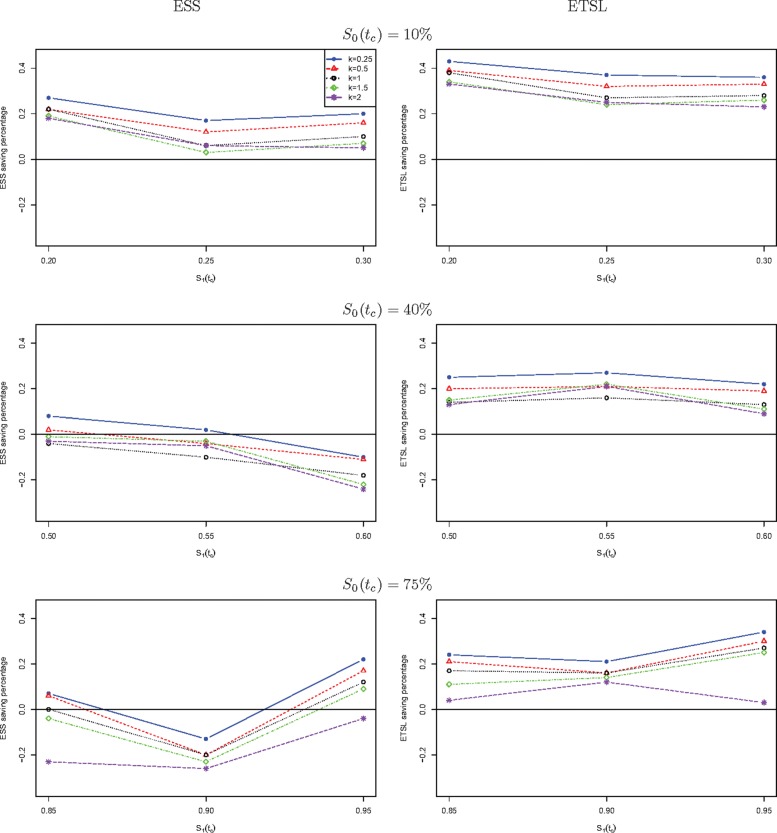
The ESS or ETSL saving of the proposed two-stage minimax design with survival endpoint as compared to Simon’s two-stage minimax design with binary endpoint when *α*=5*%* and *β*=10*%*

**Fig. 2:**
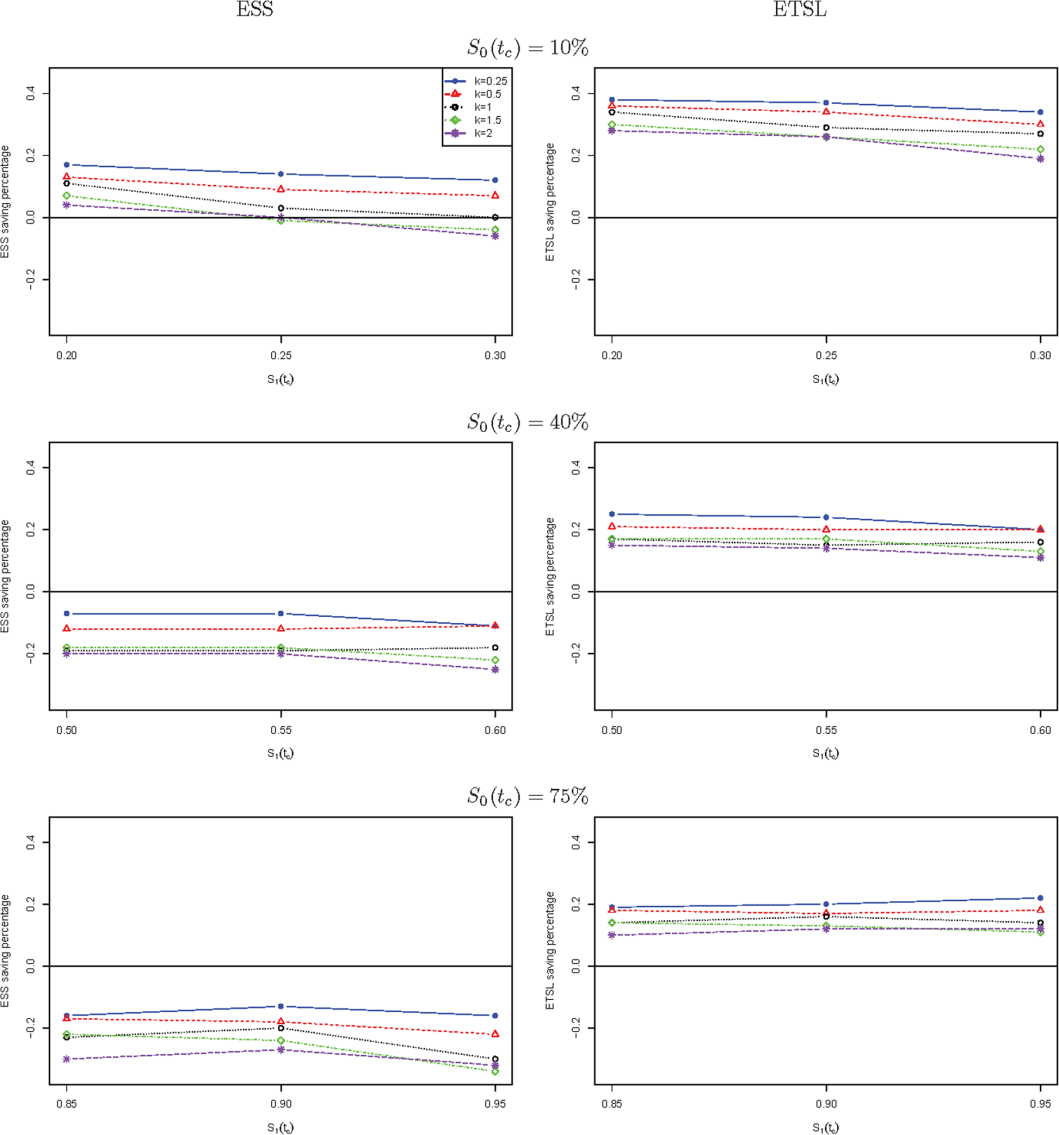
The ESS or ETSL saving of the proposed two-stage optimal design with survival endpoint as compared to Simon’s two-stage optimal design with binary endpoint when *α*=5*%* and *β*=10*%*

**Fig. 3:**
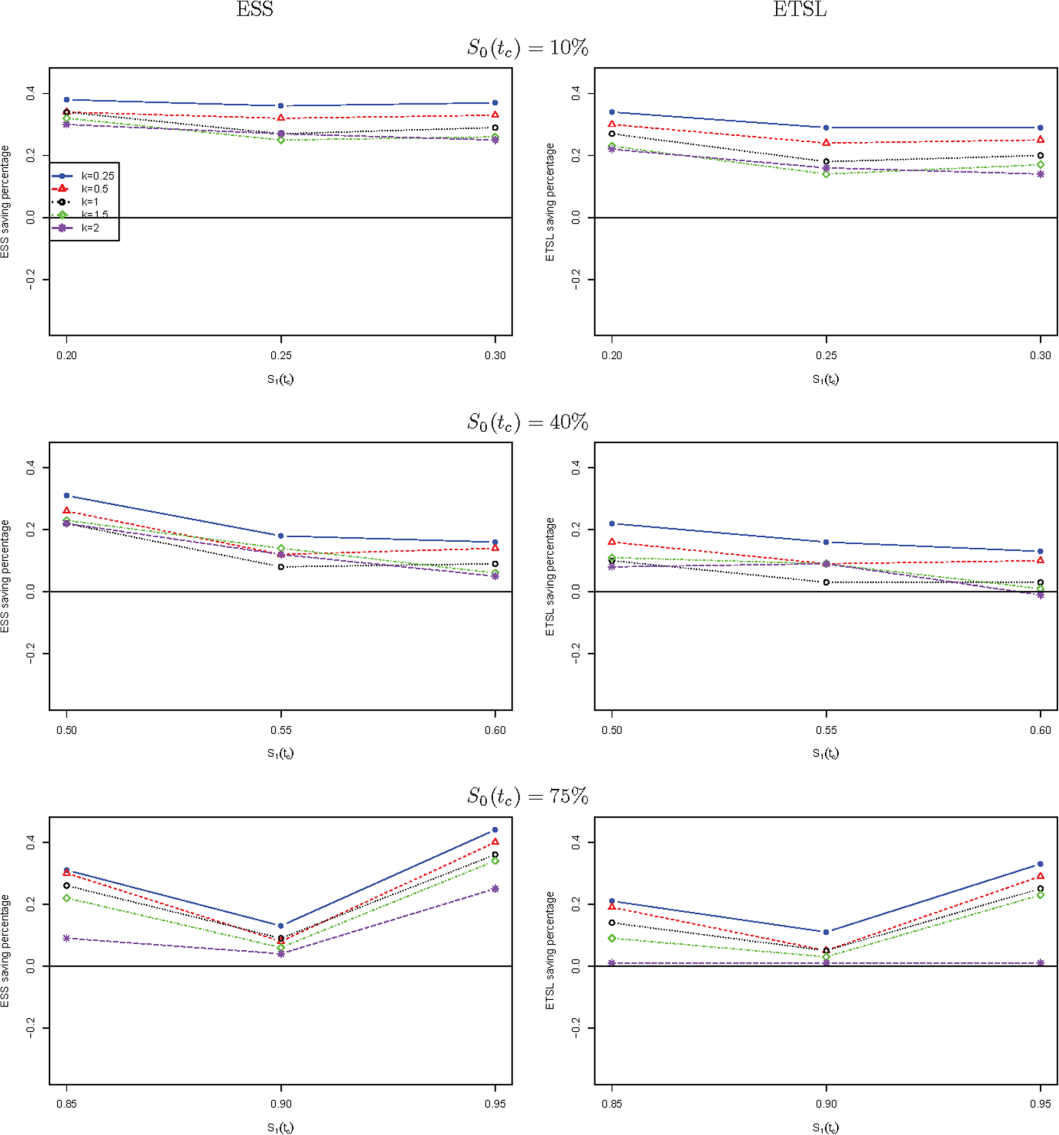
The ESS or ETSL saving of the proposed two-stage minimax design with survival endpoint as compared to Simon’s two-stage minimax design with interim accrual with binary endpoint when *α*=5*%* and *β*=10*%*

**Fig. 4:**
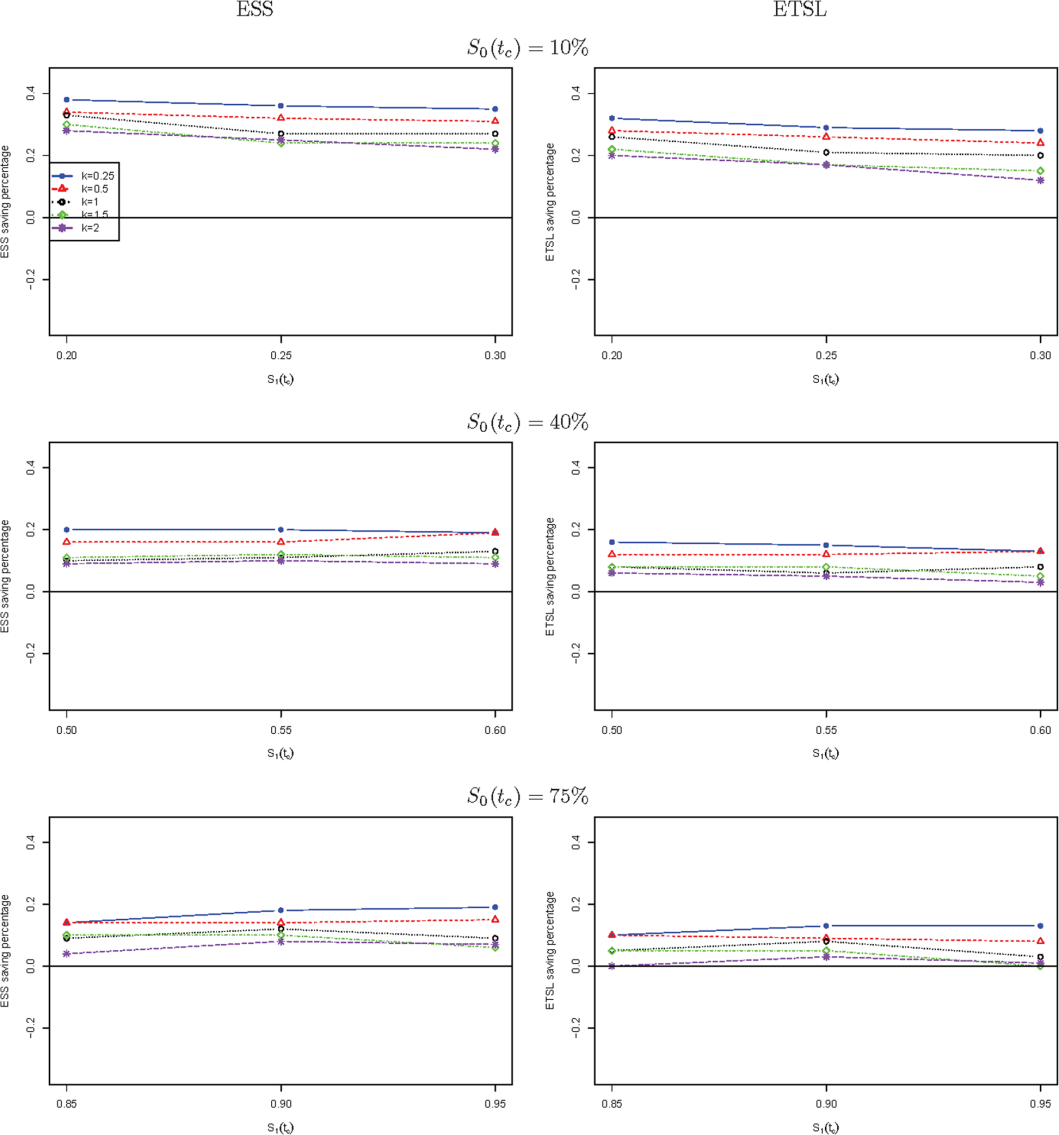
The ESS or ETSL saving of the proposed two-stage optimal design with survival endpoint as compared to Simon’s two-stage optimal design with interim accrual with binary endpoint when *α*=5*%* and *β*=10*%*

We further compare the new two-stage minimax design with Simon’s two-stage minimax design with the shape parameter *k* from 0.25 to 2 in Fig. 1 for a trial to attain 90% power at the significance level of 5%. When *S*_0_(*t*_*c*_) is low, the new design needs a smaller expected sample size than Simon’s minimax design, and this trend is reversed when *S*_0_(*t*_*c*_) is high, e.g., 40%, and 75%. The saving of the new design often decreases as *k* goes up. The new design always requires a shorter expected total study length than Simon’s minimax design. Similar results are observed in Fig. 2 where the new two-stage optimal design is compared with Simon’s optimal design. We also compare the new design with Simon’s two-stage minimax and optimal designs with interim accrual in Fig. 3 and Fig. 4, respectively. The results indicate that the new design performs better than Simon’s design with interim accrual with regards to both *ESS*_0_ and *ETSL*_0_.

### Examples

We revisit the cancer trial discussed by Case and Morgan [9] in “Simon’s two-stage designs with binary endpoint” subsection to investigate the effectiveness of a combination of Gemcitabine and external beam radiation for patients with resectable pancreatic cancer. The clinically meaningful follow-up time is assumed to be 1 year, *t*_*c*_=1. The survival probability under the null and the alternative are *S*_0_(1)=35*%*, and *S*_1_(1)=50*%*, respectively. The survival function follows an exponential distribution. This trial is designed to attain 90% power at the significance level of 10%. We compute the detailed two-stage designs with survival endpoint, including sample sizes and critical values for each stage in Table 1. The *ESS*_0_ of the new design is slightly larger than that of Simon’s design, but much smaller than that of Simon’s design with interim accrual. The *ETSL*_0_ of the new design is always shorter than that of Simon’s designs with or without interim accrual, and the study time saving is substantial.

We also consider a second clinical trial evaluating the activity of a combination of irinotecan and cisplatin for patients with refractory or recurrent non-small cell lung cancer [20]. The response rates are 10% and 25% under the null and the alternative hypotheses. Suppose the clinically meaningful follow-up time is 1 year. For Simon’s two-stage optimal design when *α*=5*%* and *β*=20*%*, the maximum possible sample size is *n*=43 and the expected sample size under the null hypothesis is *ESS*_0_=24.7, see Table 5 for the case with *S*_0_(*t*_*c*_)=10*%* and *S*_1_(*t*_*c*_)=25*%*. The proposed new two-stage optimal design with survival endpoint needs a slightly smaller *ESS*_0_ as 24.0, and can save the expected total study length by almost 1 year (2.2 VS 3.1 from Simon’s design). A 95% two-sided confidence interval of the response rate was reported in the original research article by Takiguchi et al. [20]. The hypothesis is one sided in both Simon’s design and the proposed design. Therefore, a 90% two-sided confidence interval for the response rate or the survival rate should be reported when *α*=5*%*.

## Discussion

In the design search process, we search for the minimax and optimal designs when both designs have power above the nominal level. In practice, when one type of design is of interest (e.g., the two-stage minimax design), we would suggest searching for the design such that power of this particular type design is above the nominal level. The written R program computes the designs to have both the minimax design and the optimal design meet the nominal power level, which is available upon request from the first author.

## Conclusions

The commonly used Simon’s two-stage design has to suspend the enrollment temporally after *n*_1_ patients enrolled in the first stage [5, 11, 21–28]. The research team has to wait a while (*t*_*c*_) until all *n*_1_ patients complete the study. The calculated test statistic from the first stage is then compared to the pre-determined critical value to make a go or no-go decision to the second stage. Meanwhile, the proposed two-stage designs with survival endpoint do not have to suspend the trial, thus the comparison between the proposed design with Simon’s two-stage design with no interim accrual is not very appropriate. Due to the popularity of Simon’s two-stage design, we include this design as reference. Simon’s two-stage design with interim accrual is a reasonable competitor for the proposed two-stage design with survival endpoint.

## Appendix

## Test statistics of *Z*_1_ and *Z*

At the end of the first stage *t*_1_, the observed time for the *i*-th patient is *O*_*i*_= min(*T*_*i*_,*C*_*i*_, max(0,*t*_1_−*τ*_*i*_)), where *C*_*i*_=*t*_*c*_ with restricted follow-up, and *i*=1,2,⋯,*n*_1_. Let *N*_*i*_(*t*)=*I*(*T*_*i*_≤ min(*C*_*i*_, max(0,*t*−*τ*_*i*_)))*I*(*T*_*i*_≤*t*) and *Y*_*i*_(*t*)=*I*(*T*_*i*_≥*t*,*T*_*i*_≥*t*_*c*_) be the event process and the at-risk process, respectively. The one-sample log-rank test at the end of the first stage is expressed as:

$$Z_{1}=\frac{O-E}{\sqrt{E}}, $$

where $O=\sum _{i=1}^{n} \int _{0}^{\infty } d N_{i}(t)$ are $E=\sum _{i=1}^{n} \int _{0}^{\infty } Y_{i} (t) d \Lambda _{0}(t)$ are the observed number of events and the expected number of events, respectively. The one-sample log-rank test can be alternatively written as

$$Z_{1}=\frac{W_{1}}{\hat\sigma_{1}}, $$

where $W_{1}=(O-E)/\sqrt {n}$ and $\hat \sigma =E/n$, and $\hat \sigma _{1}^{2}$ is the variance estimate of *W*_1_. The one-sample log-rank test *Z* at the end fo the study can be derived similarly by replacing *N*_*i*_(*t*) with *N*_*i*_(*t*)=*I*(*T*_*i*_≤*C*_*i*_)*I*(*T*_*i*_≤*t*).

## Mean and variance estimates of *W*_1_ and *W* under the null hypothesis

The mean of *W*_1_ or *W* under the null hypothesis is 0. The clinically meaningful follow-up time *t*_*c*_ is the upper bound follow-up time for each patient, then the censoring distribution is *G*(*t*)=*I*(*t*≤*t*_*c*_). The censoring distribution for the first stage is *G*_1_(*t*)=*U*(0,*t*_1_)*I*(*t*≤*t*_*c*_) due to a possible short follow-up time at the data analysis time *t*_1_. Then, the variances of *W*_1_ and *W* are estimated as

$${\begin{aligned} \sigma_{01}^2=Var(W_{1})=-\int_{0}^{t_{c}} G_{1}(t)d S_{0}(t)\ \text{and} \\ \ \sigma_{02}^2=Var(W)=-\int_{0}^{t_{c}} G(t)d S_{0}(t). \end{aligned}} $$

It follows that the correlation between *W*_1_ and *W* under *H*_0_ is *ρ*_0_=*σ*_01_/*σ*_02_. The TIE in Eq. (3) can then be computed after the correlation coefficient *ρ*_0_ being estimated.

## Mean and variance estimates of *W*_1_ and *W* under the alternative hypothesis

Under the alternative hypothesis, the mean values of *W*_1_ and *W* are

$$E(W_{1})=\sqrt{n_{1}} \omega_{1}\ \text{and} \ \ E(W)=\sqrt{n} \omega $$

where *ω*=*p*_1_−*p*_0_, $p_{1}=\int _{0}^{t_{c}} G(t)S_{1}(t) d \Lambda _{1}(t)$, $p_{0}=\int _{0}^{t_{c}} G(t)S_{1}(t) d \Lambda _{0}(t)$, and *ω*_1_=*p*_1*f*_−*p*_0*f*_, $p_{1f}=\int _{0}^{t_{c}} G_{1}(t)S_{1}(t) d \Lambda _{1}(t)$, $p_{0f}=\int _{0}^{t_{c}} G_{1}(t)S_{1}(t) d \Lambda _{0}(t)$. Recently, Wu [17] derived the exact variance of *W* under the alternative hypothesis as

$$\sigma_{12}^2=Var(W)=p_{1}-p_{1}^2-p_{0}^2+2p_{0} p_{1} +2 p_{00}-2 p_{01}, $$

where $p_{00}=\int _{0}^{t_{c}} G(t)S_{1}(t) \Lambda _{0}(t) d \Lambda _{0}(t)$ and $p_{01}=\int _{0}^{t_c} G(t)S_{1}(t) \Lambda _{0}(t) d \Lambda _{1}(t)$. The exact variance of *W*_1_, $\sigma _{11}^2=Var(W_{1})$, can be derived similarly. It follows that the correlation between *W*_1_ and *W* under *H*_1_ is *ρ*_1_=*σ*_11_/*σ*_12_, and power of a two-stage design is

4 $$ Power=\int_{-\infty}^{\tilde{c}} \phi(t) \Phi\left(\frac{\tilde{c_{1}}-\rho_{1} t}{\sqrt{1-\rho_{1}^{2}}}\right) d t,   $$

where $\tilde {c_{1}}=\frac {\sigma _{01}}{\sigma _{11}}\left (c_{1}-\frac {\omega _{1} \sqrt {n_{1}}}{\sigma _{01}}\right)$, and $\tilde {c}=\frac {\sigma _{02}}{\sigma _{12}}\left (c-\frac {\omega _{2} \sqrt {n_{2}}}{\sigma _{02}}\right)$.

## Abbreviations

ESS: Expected sample size
ETSL: Expected total study length
PET: Probability of early termination
TIE: Type I error
